# Retraction: p53 loses grip on PIK3CA expression leading to enhanced cell survival during platinum resistance

**DOI:** 10.1002/1878-0261.13615

**Published:** 2024-03-04

## Abstract

The above article, published online on 29 April 2016 in Wiley Online Library (wileyonlinelibrary.com), has been retracted by agreement between the journal Editor‐in‐Chief, Kevin Ryan, FEBS Press, and John Wiley and Sons Ltd. The retraction has been agreed due to several image duplications in Figures 4B,E,G, and 5C,D, and inconsistencies between tumour luminescence images and the quantification in Figure 4K. Compelling raw data images were not available from the authors. The editors consider the conclusions substantially compromised and are therefore retracting the paper. Corresponding author Pritha Ray agrees to this retraction, since the raw data are no longer available for analysis and validation.

Reference

1 Thakur B, Ray P. p53 loses grip on PIK3CA expression leading to enhanced cell survival during platinum resistance. *Mol Oncol*. 2016;10(8):1283–1295. https://doi.org/10.1016/j.molonc.2016.06.006

The above article, published online on 29 April 2016 in Wiley Online Library (wileyonlinelibrary.com), has been retracted by agreement between the journal Editor‐in‐Chief, Kevin Ryan, FEBS Press, and John Wiley and Sons Ltd. The retraction has been agreed due to several image duplications in Figures 4B,E,G, and 5C,D, and inconsistencies between tumour luminescence images and the quantification in Figure 4K. Compelling raw data images were not available from the authors. The editors consider the conclusions substantially compromised and are therefore retracting the paper. Corresponding author Pritha Ray agrees to this retraction, since the raw data are no longer available for analysis and validation.

**Reference**

1 Thakur B, Ray P. p53 loses grip on PIK3CA expression leading to enhanced cell survival during platinum resistance. *Mol Oncol*. 2016;10(8):1283–1295. https://doi.org/10.1016/j.molonc.2016.06.006

